# The Left Axillary Artery as an Alternative Inflow Source in Minimally Invasive Coronary Artery Bypass Grafting: Safety, Feasibility, and Mid-Term Outcomes

**DOI:** 10.3390/jcdd13020101

**Published:** 2026-02-21

**Authors:** Jian Song, Tong Ding, Rui Li, Yichen Gong, Ruitao Zhang, Yuanhao Fu, Luyu Meng, Song Wu, Zhongqi Cui, Ya Wu, Chen Yang, Ming Cui, Yunpeng Ling

**Affiliations:** 1 Department of Cardiac Surgery, Peking University Third Hospital, Peking University Health Science Center, Beijing 100191, China; 2 Department of Cardiology, Peking University Third Hospital, Peking University Health Science Center, Beijing 100191, China

**Keywords:** axillary artery, saphenous vein graft, proximal anastomosis, graft inflow source, MICS-CABG

## Abstract

Objective: The objective of this study is to evaluate the safety, feasibility, and mid-term outcomes of using the left axillary artery (AXA) as an alternative inflow source for the proximal anastomosis of the saphenous vein graft (SVG) in MICS-CABG, focusing on intraoperative graft haemodynamics, early patency, and clinical outcomes. Methods: We retrospectively analyzed consecutive patients who underwent MICS-CABG between April 2020 and August 2025 at a single center. Patients were divided into two groups based on the inflow source: the ascending aorta (n = 292) or the left axillary artery (n = 90). After propensity score matching, 80 matched pairs were analyzed. Intraoperative graft haemodynamics were assessed. Early graft patency was evaluated using coronary angiography or CT angiography. Mid-term outcomes, including overall survival and major adverse cardiac and cerebrovascular events (MACCEs), were compared between groups. Results: Both groups demonstrated comparable intraoperative hemodynamic performance. The AXA group demonstrated an early graft occlusion rate comparable to that of the AOR group (1.32% vs. 3.16%, RR = 0.42, 95% CI = 0.08–2.11, and *p* = 0.45). Overall survival (93.2% vs. 100%, *p* = 0.06) and the MACCE-free metric (91.9% vs. 92.1%, *p* = 0.83) showed no significant difference between groups. Conclusions: The left axillary artery is a safe and feasible alternative inflow source in MICS-CABG. This approach provides acceptable intraoperative flow dynamics, early patency, and mid-term outcomes to conventional ascending aortic inflow.

## 1. Introduction

Minimally invasive cardiac surgery–coronary artery bypass grafting (MICS-CABG) offers the advantage of achieving standardized surgical revascularization while avoiding full sternotomy and cardiopulmonary bypass, thereby accelerating postoperative recovery and reducing surgical trauma [1,2]. However, when the ascending aorta is severely calcified, contains extensive atherosclerotic plaques, or is dilated with an increased risk of rupture after clamping, identifying an alternative arterial inflow source for proximal anastomosis of the saphenous vein graft becomes a pressing clinical challenge.

The axillary artery is a medium-sized muscular–elastic artery with a relatively thick wall and intact internal elastic lamina and media, making it well suited for suturing and for withstanding anastomotic stress. Compared with the ascending aorta, the axillary artery usually has a lower burden of calcification and atherosclerosis—especially in patients with a “porcelain” aorta or extensive aortic disease—thereby reducing the risk of embolic sources at the proximal anastomosis and of dislodging plaques during manipulation. The axillary artery was first reported as a proximal inflow source for saphenous vein grafting by Yaryura et al. in 1997 [3]. Since then, several subsequent studies have demonstrated the feasibility of this technique, suggesting favorable graft flow and patency outcomes [4,5,6,7,8]. Nevertheless, most of the studies over the past two decades were limited by case reports and narrow evaluation parameters.

In the present study, we retrospectively analyzed data from a prospectively maintained single-center MICS-CABG database to evaluate the feasibility, safety, and mid-term outcomes of using the axillary artery as the proximal inflow source, both at the graft level and the patient level.

## 2. Materials and Methods

This prospectively maintained database was conducted at Peking University Third Hospital, with institutional review board approval (IRB00006761-M2019033, 5 August 2019). All patients signed an informed written consent form to publish their study data. This study adhered to the Strengthening the Reporting of Observational Studies in Epidemiology guideline.

**Figure 1:**
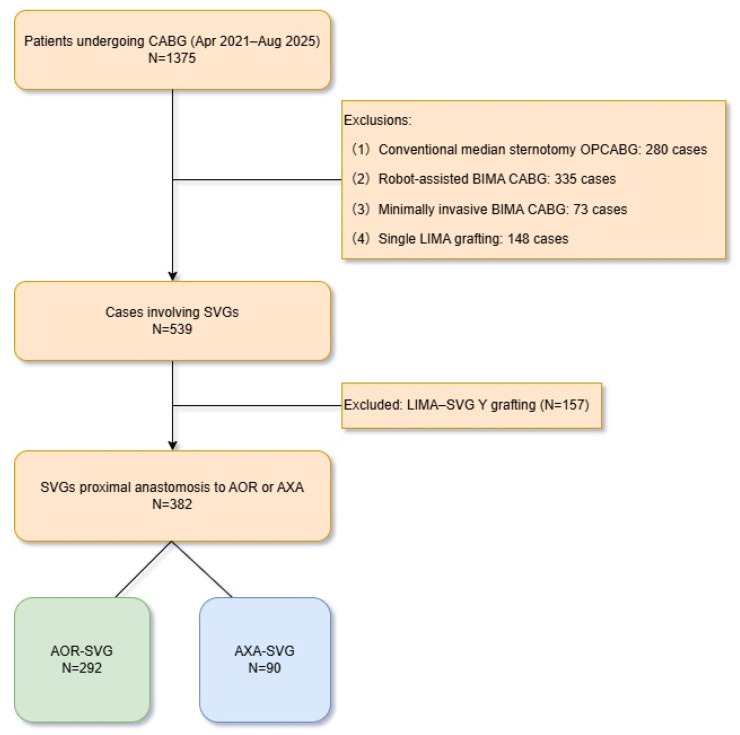
Patient screening flowchart with inclusion and exclusion criteria. OPCABG, off-pump coronary artery bypass grafting; BIMAs, bilateral internal mammary arteries; LIMA, left internal mammary artery; SVG, saphenous vein graft; AXA, axillary artery.

A retrospective analysis was performed using a prospectively maintained database covering the period from April 2020 to August 2025. Patients who met the inclusion criteria and had undergone MICS-CABG were retrospectively included in the study. The grafting strategy involved proximal anastomosis of the saphenous vein graft (SVG) to either the ascending aorta or the left axillary artery. A total of 382 consecutive patients were included. Those with the SVG proximally anastomosed to the ascending aorta were assigned to the Aorta group (AOR, n = 292), and those with the SVG anastomosed to the left axillary artery were assigned to the Axillary group (AXA, n = 90). The patient screening flowchart is provided in Figure 1.

The indications for selecting the left axillary artery as the proximal anastomosis site for saphenous vein grafts include (1) preoperative imaging demonstrating extensive calcification or atherosclerotic plaques of the ascending aorta and (2) preoperative aorta computed tomography angiography showing ascending aortic dilatation greater than 4.0 cm, without concomitant aortic valve disease and the need for simultaneous ascending aortic intervention. All patients routinely underwent preoperative and pre-discharge duplex ultrasonography of both upper extremities to assess the hemodynamics of the subclavian, axillary, radial, and ulnar arteries, as well as to detect any vascular stenosis.

### 2.1. Surgical Approach

**Figure 2:**
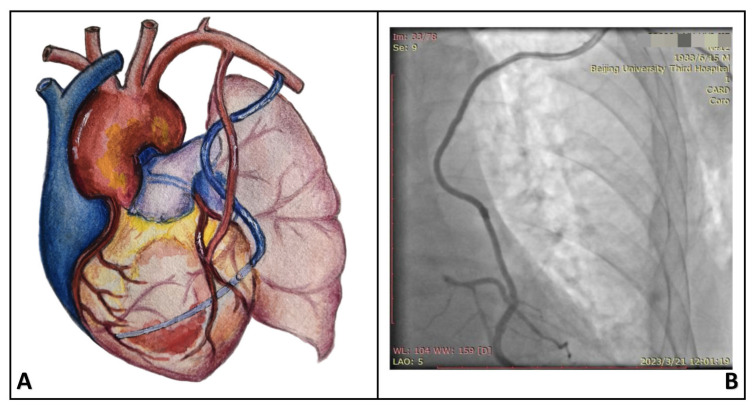
Schematic illustration of the saphenous vein graft course with proximal anastomosis to the left axillary artery (**A**), as well as the corresponding postoperative graft angiography (**B**).

MICS-CABG was performed using an off-pump technique through a minimally invasive thoracotomy (4–6 cm incision) placed in the left fourth or fifth intercostal space between the midclavicular and anterior axillary lines. All procedures were conducted under direct vision. Details on the surgical techniques have been described previously [8]. In the Aorta (AOR) group, a side-biting clamp was applied to the aortic wall, followed by an aortic punch and proximal anastomosis of the saphenous vein graft (SVG). In the Axillary (AXA) group, a 3 cm transverse incision was made below the left clavicle at the junction of the middle and lateral thirds. The left axillary artery was dissected and exposed, and the proximal end of the SVG was anastomosed to the axillary artery using 6–0 Prolene sutures. The SVG was then passed through the first intercostal space into the thoracic cavity, routed along the chest wall to the level of the aortic arch, and further routed along the left cardiac border toward the target coronary vessels for sequential distal anastomoses. Detailed procedural aspects of MICS-CABG and the inclusion and exclusion criteria relative to conventional CABG or MICS-CABG have been reported previously and are therefore not reiterated here [9]. The configuration of the SVG and its corresponding postoperative angiographic appearance in the AXA group are presented in Figure 2.

### 2.2. Graft Assessment

Transit-time flowmetry (TTFM) is an essential and standardized tool for intraoperative assessment of graft quality during CABG. In this study, TTFM was used to measure and record the mean graft flow (MGF) and pulsatility index (PI) for each anastomosis. These parameters served as indicators of the immediate intraoperative hemodynamic performance of the grafts. Within one month after surgery, all patients underwent coronary angiography or coronary computed tomography angiography (CTA) to evaluate early graft patency. CTA was performed on a 320-detector CT scanner (uCT 960+, United Imaging, Shanghai, China) using prospective ECG gating in axial scan mode with data acquisition within a single cardiac cycle. Z-axis coverage was set to 12, 14, or 16 cm depending on heart size. Images were reconstructed on a 512 × 512 matrix with a gantry rotation time of 0.25 s, as well as a slice thickness and interval of 0.5 mm. Data were acquired during 30–80% of the cardiac cycle, with ePhase optimization for the best reconstruction phase and the use of CardioCapture technology to suppress coronary motion artifacts. Graft occlusion was defined as the lack of contrast filling within the graft, abrupt interruption of contrast flow, or a thread-like appearance on angiography or CTA.

### 2.3. Outcome Definitions

The primary outcome was defined as graft patency evaluated by early coronary angiography or CTA, reflecting outcomes at the graft level. The secondary outcomes included perioperative mortality, stroke, new-onset myocardial infarction (MI), and major adverse cardiovascular and cerebrovascular events (MACCEs) during follow-up, defined as a composite of death, MI, stroke, and target vessel ischemia-driven repeat revascularization. The occurrence of shoulder, elbow, or wrist motion impairment, or upper-limb sensory abnormalities, was defined as axillary artery-related complications.

### 2.4. Follow-Up

All patients were followed postoperatively through outpatient visits, WeChat, or telephone contact. Key follow-up time points were at 1, 3, and 6 months and 1 year after discharge, with annual follow-up thereafter. Follow-up assessments included survival status, repeat revascularization and stroke assessments. Ultimately, only 5 patients were lost during follow-up, corresponding to an overall loss-to-follow-up rate of 1.3%. The median follow-up duration was 28.5 (13.3–46.0) months.

### 2.5. Statistical Analysis

Statistical analysis and data visualization were performed using IBM SPSS Statistics (Version 26.0; IBM Corp., Armonk, NY, USA). Continuous variables were first tested for normality. Normally distributed data are presented as the mean ± standard deviation (SD) and were compared between groups using Student’s *t*-test. Non-normally distributed data are expressed as the median with the interquartile range (IQR) and were compared using the Mann–Whitney U test. Categorical variables are presented as frequencies and percentages and were analyzed using the chi-square test or Fisher’s exact test, as appropriate. All statistical tests were two-tailed, and a *p*-value of < 0.05 was considered statistically significant.

To adjust for unbalanced basic characteristics, a propensity score matching (PSM) analysis was performed at a 1:1 ratio with a nearest neighbor-matching algorithm. The preoperative variables (including sex, age, BMI, diabetes, hyperlipemia, hypertension, stroke, smoking, chronic kidney disease, percutaneous coronary intervention, myocardial infarction, New York Heart Association Functional Classification III-IV, serum creatinine, total cholesterol, low-density lipoprotein cholesterol, left ventricular ejection fraction, and left ventricular end-diastolic diameter) in the two groups were performed with PSM, and the *p*-values showed no statistically significant differences, and the standardized mean differences (SMDs) for all variables were controlled below 0.2. Then, the perioperative variables were compared between the groups.

Survival over time and freedom from MACCEs were estimated by the Kaplan–Meier curve, with hypothesis testing performed using the log-rank test after matching. Kaplan–Meier curves were also generated to illustrate the actual follow-up outcomes between the two unmatched groups, with selected baseline variables adjusted as covariates in a Cox proportional hazards model.

## 3. Results

A total of 382 patients were included in the study cohort, with a mean age of 66.3 years, and 71.5% were male. Before matching, patients in the AXA group were significantly older (71.0 (65.0–77.3) years vs. 67.0 (61.0–62.0) years, *p* < 0.001) and had a higher proportion of NYHA III–IV heart failure (43.3% vs. 21.9%, *p* < 0.001). Seventeen preoperative baseline variables were included in the PSM analysis, which was performed at a 1:1 ratio. A total of 80 matched pairs (309 saphenous vein grafts) who all had early postoperative imaging data were successfully generated. After matching, no significant differences were observed between the two groups at the patient level. The baseline characteristics of patients before and after PSM are summarized in Table 1, and the love plot is shown in Supplementary Figure S1.

### 3.1. Graft-Level Analysis

All patients received a left internal mammary artery (LIMA) graft to the left anterior descending artery (LAD). In addition, there was no significant difference in the number of SVGs between the two groups (2.0 (1.0–2.5) vs. 2.0 (1.0–2.0), *p* = 0.43). The distal anastomoses of SVGs were performed in a sequential anastomosis to revascularize the left circumflex (LCX) and right coronary artery (RCA) systems.

**Figure 3:**
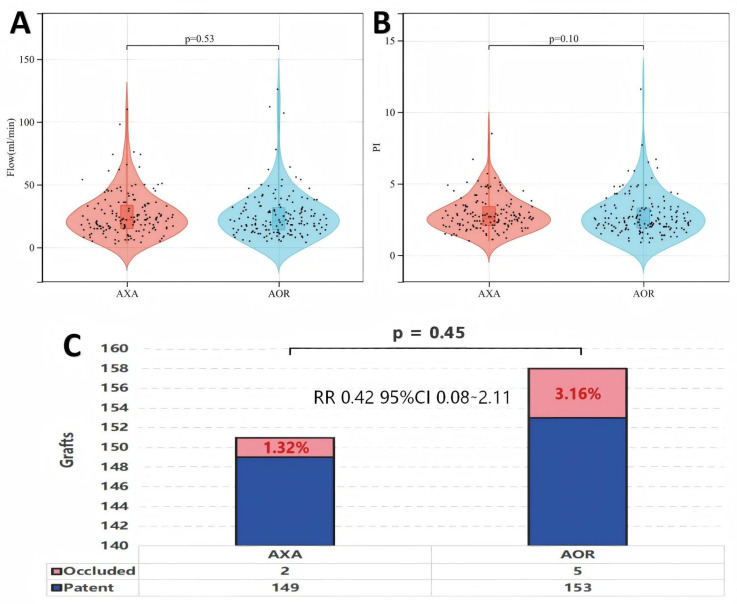
Intraoperative graft-level hemodynamics and early postoperative outcomes. (**A**) Comparison of mean flow measured by transit-time flowmetry (TTFM) between the two groups. (**B**) Comparison of the pulsatility index (PI) between the two groups. (**C**) Early graft occlusion rates between the two groups. AOR, Aorta group; AXA, Axillary group.

Intraoperative TTFM measurements showed no significant differences in graft flow or PI between the two groups (AXA vs. AOR: Flow, 22 (15–34) mL/min vs. 21 (13–32) mL/min, *p* = 0.53; PI, 2.6 (1.9–3.3) vs. 2.4 (2.1–3.4), *p* = 0.10) (Figure 3A,B).

Early postoperative graft patency was evaluated in 125 patients who underwent coronary angiography and 35 patients who underwent CTA. The AXA group demonstrated an early graft occlusion rate comparable to that of the AOR group (RR = 0.42 and 95% CI = 0.08–2.11, *p* = 0.45). In the AXA group, two SVGs were occluded (one proximal to the obtuse marginal (OM) anastomosis and one proximal to the posterolateral (PL) anastomosis), resulting in an occlusion rate of 1.32%. In the AOR group, five SVGs were occluded (three proximal to the posterior descending artery (PDA) anastomosis and two proximal to the OM anastomosis), corresponding to an occlusion rate of 3.16% (Figure 3C).

### 3.2. Patient-Level

The operative time was significantly longer in the AXA group (281.8 ± 64.5 min vs. 255.6 ± 67.4 min, *p* = 0.02). There were no perioperative deaths in the AOR group, whereas one patient in the AXA group experienced perioperative mortality. In the AXA group, one patient developed clinical signs of left brachial plexus injury before discharge, presenting with shoulder joint weakness and numbness in the upper limb, and was treated with neurotrophic therapy and rehabilitation. The detailed intraoperative and postoperative data of the two groups are presented in Table 2.

**Figure 4:**
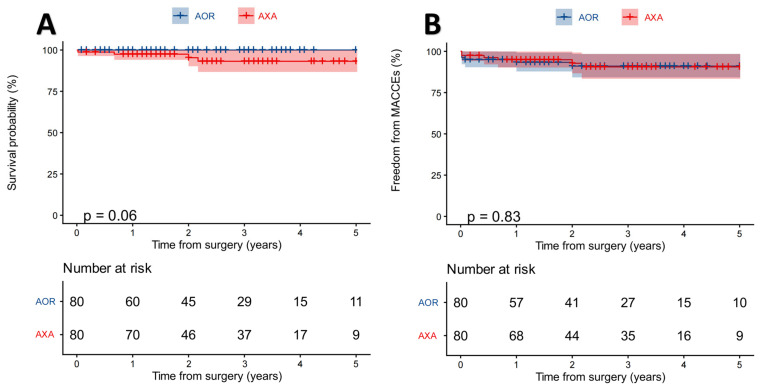
Kaplan–Meier curves for mid-term follow-up outcomes after PSM. (**A**) Overall survival curve; (**B**) freedom from MACCEs. The shaded areas represent 95% confidence intervals. The *p*-values were calculated using the log-rank test. AOR, Aorta group; AXA, Axillary group.

Kaplan–Meier survival analysis showed that the 1-year cumulative survival rate was 97.5% (CI: 93–100%) in the AXA group and 100% (CI: 100–100%) in the AOR group, while the 3-year cumulative survival rate was 93.2% in the AXA group and 100% in the AOR group. The log-rank test had a value of *p* = 0.06 (Figure 4A). Three deaths occurred in the AXA group during the follow-up period: one patient died from COVID-19-related complications at 8 months postoperatively, and two patients died of cardiac causes at 24 and 26 months, respectively.

The stroke-free survival rate at 1 and 3 years was 100% in the AXA group and 97.1% in the AOR group, with two cases of cerebral infarction occurring in the AOR group at 30 days and 1 year postoperatively, respectively. The freedom from MACCEs was slightly higher in the AXA group during the early postoperative period (96.2%, CI: 91.1–98.4% vs. 92.1%, CI: 90.1–98.4%), but there was no significant difference in the mid-term follow-up (*p* = 0.83) (Figure 4B).

## 4. Discussion

The intraoperative hemodynamic performance at the graft level was comparable between cases with SVG proximal anastomosis to the ascending aorta and those to the left axillary artery. The AXA group demonstrated a trend toward better early graft patency. At the patient level, there were no differences in mid-term survival or the incidence of MACCEs between the two groups.

MICS-CABG can substantially reduce surgical trauma and improve early postoperative quality of life while ensuring complete surgical revascularization [1,10,11,12,13]. However, when the ascending aorta is heavily calcified or slightly dilated, determining a suitable site for the proximal graft anastomosis becomes a major challenge. The axillary artery can serve as an alternative inflow source to overcome this limitation, with an operative approach that is largely consistent with that of MICS-CABG [3]. In 2001, Coulson et al. systematically summarized the early international experience of using the axillary artery as an inflow source, with reported graft patency rates of 70–100% and no major complications [14]. Even in multivessel totally endoscopic coronary artery bypass grafting (TECAB), the axillary artery has been reported to yield favorable outcomes in individual cases [15]. In the present study, the operative mortality in the AXA group before matching was 1.1%, the early patency rate was 98.7%, and the intraoperative flow and PI values were comparable to those observed with the conventional proximal anastomosis to the ascending aorta. Notably, the early graft patency rate in the AXA group was slightly higher than that in the AOR group, which is consistent with the recent findings reported by Ushioda et al. [7].

Linden et al. demonstrated that the incidence of postoperative stroke was significantly lower in patients without atherosclerotic plaques in the ascending aorta compared with those with aortic atheroma (1.8% vs. 8.7%, *p* < 0.001) [16]. Furthermore, a large meta-analysis reported that anaortic CABG was associated with a substantially reduced risk of stroke compared with partial aortic clamping (OR: 0.34; 95% CI: 0.22–0.52) [17]. In this cohort, nine cases of cerebral infarction occurred in the AOR group during follow-up before matching, and two cases remained even after propensity matching, whereas no stroke events were observed in the AXA group (0/90, 0.0%; 95% Clopper–Pearson CI = 0.0–3.3%). The curves describing overall survival and freedom from MACCEs for the original unmatched cohort are presented in Supplementary Figure S2. Consequently, avoiding manipulation of a calcified or atherosclerotic ascending aorta is crucial for reducing perioperative stroke risk. In this context, the axillary artery provides a reliable and physiologically favorable alternative inflow source for proximal graft anastomosis. In addition, there is another specific scenario where the axillary artery anastomosis is particularly applicable in MICS-CABG. When the ascending aorta is rightwardly displaced or rotated, exposure and clamping of the aorta through a left anterolateral mini-thoracotomy become technically challenging. In such cases, using the axillary artery as the proximal anastomosis site offers a practical and safe alternative, especially for surgeons who do not have much experience in MICS-CABG.

In summary, we propose the following primary clinical indications for selecting the left axillary artery as the proximal anastomotic strategy: (1) preoperative imaging demonstrating severe calcification of the ascending aorta or extensive atherosclerotic plaque burden; (2) mild dilation of the ascending aorta (diameter > 4.0 cm but not meeting the criteria for intervention); and (3) rightward (dextral) rotation of the ascending aorta that precludes safe clamping and exposure.

After the SVG is anastomosed proximally to the axillary artery, the graft must traverse a relatively long course, passing through the intercostal muscles and the thoracic cavity [18]. The potential impact of this course on graft patency was a major concern. Reassuringly, no such risks or adverse events occurred in our series. Based on our institutional experience, several technical considerations are critical to ensure optimal graft quality: (1) The orientation of the proximal anastomosis is essential. Directing the graft toward the right anteroinferior direction provides a physiologically favorable trajectory for the subsequent course. (2) The intercostal muscles should be fully dissected, creating sufficient space to avoid compression of the SVG. (3) Once the graft enters the thoracic cavity, it should course anteromedially along the thoracic wall and into the mediastinum, with an appropriate length to prevent twisting or kinking. Collectively, these technical details are key to achieving excellent graft quality when anastomosing the SVG to the axillary artery.

### Study Limitations

This study has several limitations. It is a single-center cohort with a relatively limited sample size, which may prevent certain differences from reaching statistical significance. In addition, graft-level outcomes were evaluated only in the early postoperative phase, lacking mid- to long-term angiographic or CTA follow-up data. From a statistical perspective, graft occlusion occurred in 7 grafts from 7 patients among 309 grafts in 160 patients, indicating minimal intra-patient clustering. Given this extremely low event rate, mixed-effects or generalized estimating equation models would likely be unstable. For this reason, Fisher’s exact test was considered the most appropriate method for rare-event comparison. This limitation should be considered when interpreting graft-level analyses. Moreover, although propensity score matching incorporating multiple preoperative baseline variables was performed to minimize bias, it may not completely account for the specific clinical characteristics of patients in the axillary artery group. When confronted with unfavorable ascending aortic anatomy, the selection of the proximal anastomosis strategy is inevitably influenced by the operating surgeon’s experience and position on the learning curve. This may also introduce a slight bias into the results of the present study. Therefore, larger multi-center prospective studies with longer follow-up are warranted to further validate the safety and efficacy of using the axillary artery as an inflow source and to provide stronger evidence for clinical decision-making.

## 5. Conclusions

The left axillary artery was associated with acceptable flow characteristics, early graft patency, and favorable mid-term outcomes for saphenous vein grafts in MICS-CABG and appears particularly well suited to carefully selected patients with hostile ascending aortic anatomy.

## Figures and Tables

**Table 1:** Baseline characteristics before and after propensity score matching.

	Before Matching				After Matching			
	AOR Group (n = 292)	AXA Group (n = 90)	*p*	SMD	AOR Group (n = 80)	AXA Group (n = 80)	*p*	SMD
Sex, male (n, %)	202 (69.2)	71 (78.9)	0.07	0.223	60 (75.0)	61 (71.3)	0.85	0.029
Age, years	67.0 (61.0–72.0)	71.0 (65.0–77.3)	<0.001	0.646	69.9 ± 6.9	70.4 ± 7.5	0.66	0.069
BMI	24.6 (22.8–26.7)	24.2 (22.8–26.2)	0.34	0.123	24.9 ± 3.3	24.7 ± 3.3	0.69	0.064
Diabetes (n, %)	136 (46.7)	45 (50.0)	0.59	0.069	38 (47.5)	37 (46.3)	0.87	0.025
Hyperlipemia (n, %)	84 (28.9)	18 (20.0)	0.10	0.205	15 (18.8)	17 (21.3)	0.69	0.063
Hypertension (n, %)	180 (61.9)	63 (70.0)	0.16	0.177	58 (72.5)	56 (70.0)	0.72	0.055
Stroke (n, %)	49 (16.8)	22 (24.4)	0.11	0.190	20 (25.0)	18 (22.5)	0.71	0.059
Smoking (n, %)	96 (33.1)	37 (41.1)	0.16	0.171	33 (41.3)	29 (36.3)	0.52	0.103
CKD (n, %)	10 (3.4)	9 (10.0)	0.02	0.265	4 (5.0)	6 (7.5)	0.51	0.103
PCI (n, %)	42 (14.4)	15 (16.7)	0.60	0.063	12 (15.0)	11 (13.8)	0.82	0.036
MI (n, %)	63 (21.6)	23 (25.6)	0.44	0.094	15 (18.8)	20 (25.0)	0.34	0.152
NYHA III or IV (n, %)	64 (21.9)	39 (43.3)	<0.001	0.469	37 (46.3)	34 (42.5)	0.63	0.076
Cr, umol/L	83 (70–95)	80 (68–101)	0.81	0.208	80 (70–95)	70 (65–77)	0.81	0.108
Tc, mmol/L	3.4 (2.7–4.2)	3.3 (2.8–3.9)	0.51	0.126	3.5 ± 0.9	3.5 ± 1.0	0.88	0.011
LDL, mmol/L	1.8 (1.4–2.4)	1.7 (1.5–2.3)	0.53	0.038	1.8 ± 0.6	2.0 ± 0.9	0.56	0.092
LVEF, %	66 (55–70)	66 (52–70)	0.82	0.081	67 (56–70)	67 (52–71)	0.80	0.027
LVEDD, mm	48.2 (45.3–52.0)	48.9 (45.5–53.0)	0.42	0.178	49.6 (45.9–52.0)	49.0 (45.6–53.0)	0.95	0.072

Values are expressed as n (%), the mean ± SD or the median (IQR) as appropriate. CKD, chronic kidney disease; PCI, percutaneous coronary intervention; MI, myocardial infarction; NYHA, New York Heart Association Functional Classification; Cr, creatinine; Tc, total cholesterol; LDL, low-density lipoprotein; LVEF, left ventricular ejection fraction; LVEDD, left ventricular end-diastolic diameter.

**Table 2:** The intraoperative and postoperative conditions after matching.

	Before Matching			After Matching		
	AOR Group (n = 292)	AXA Group (n = 90)	*p*	AOR Group (n = 80)	AXA Group (n = 80)	*p*
Operation time (min)	256.1 ± 70.0	280.7 ± 65.6	<0.001	255.6 ± 67.4	281.8 ± 64.5	0.02
Revascularization strategy						
Total counts of grafts, n (IQR)	3.0 (2.0–3.3)	3.0 (2.0–3.0)	0.35	3.0 (2.0–3.5)	3.0 (2.0–3.0)	0.52
Counts of LIMA grafts, n (IQR)	1	1	>0.99	1	1	>0.99
Counts of SVGs, n (IQR)	2.0 (1.0–3.0)	2.0 (1.0–2.0)	0.33	2.0 (1.0–2.5)	2.0 (1.0–2.0)	0.43
Intra-IABP, n (%)	2 (0.7)	3 (3.3)	0.05	1 (1.3)	3 (3.8)	0.31
Intra-RBC transfusion (U)	0	0	0.20	0	0	0.54
Perioperative death, n (%)	0	1 (1.1)	0.07	0	1 (1.3)	0.32
Perioperative MI, n (%)	5 (1.7)	2 (2.2)	0.74	2 (2.5)	2 (2.5)	>0.99
Requiring reoperation, n (%)	7 (2.4)	4 (4.4)	0.31	3 (3.80)	3 (3.8)	>0.99
POAF, n (%)	15 (5.1)	9 (10.0)	0.10	2 (2.5)	6 (7.5)	0.15
CRRT, n (%)	1 (0.3)	2 (2.2)	0.08	0	2 (2.5)	0.16
Brachial plexus injury, n (%)	0	1 (1.1)	0.07	0	1 (1.3)	0.32
Drainage in 72 h (mL)	920 (730–1250)	940 (605–1165)	0.42	970 (690–1300)	920 (605–1130)	0.29
ICU time (h)	23.0 (19.0–40.0)	21.5 (18.0–42.8)	0.81	23.0 (19.0–26.0)	21.0 (18.0–40.8)	0.55
Mechanical ventilation (h)	9.0 (6.0–14.0)	9.5 (6.0–16.0)	0.66	9.0 (6.0–13.0)	9.5 (6.0–14.8)	0.88
Post-RBC transfusion (U)	0	0	0.07	0	0	0.63
Post-cTnT (ng/mL)	0.27 (0.16–0.45)	0.35 (0.20–0.64)	0.04	0.26 (0.16–0.46)	0.35 (0.20–0.67)	0.16
Post-CKMB (U/L)	17.0 (12.0–24.0)	20.5 (14.3–33.0)	0.01	18.0 (12.5–28.0)	20.5 (15.0–33.0)	0.24
Post-BNP (pg/mL)	1545 (832–2862)	2107 (967–4372)	0.05	1834 (821–1365)	1867 (887–3771)	0.56

Values are expressed as n (%), the mean ± SD or the median (IQR) as appropriate. LIMA, left internal mammary artery; SVG, saphenous vein graft; IABP, intra-aortic balloon pump; RBC, red blood cell; MI, myocardial infarction; POAF, postoperative atrial fibrillation; CRRT, continuous renal replacement therapy; ICU, intensive care unit.

## Data Availability

The data underlying this article will be shared upon reasonable request to the corresponding authors.

## Supplementary Materials

The following supporting information can be downloaded at https://www.mdpi.com/article/10.3390/jcdd13020101/s1, Supplementary Figure S1. Distribution of standardized mean differences for baseline variables before and after propensity score matching. Supplementary Figure S2. Kaplan–Meier curves for mid-term follow-up outcomes before PSM.

## Abbreviations

AOR	ascending aorta
AXA	axillary artery
SVG	saphenous vein graft
MICS-CABG	minimally invasive cardiac surgery–coronary artery bypass grafting
PSM	propensity score matching
TTFM	transit-time flowmetry
MGF	mean graft flow
PI	pulsatility index
CTA	computed tomography angiography
MI	myocardial infarction
MACCEs	major adverse cardiovascular and cerebrovascular events
