# Supplementary material for: The Left Axillary Artery as an Alternative Inflow Source in Minimally Invasive Coronary Artery Bypass Grafting: Safety, Feasibility, and Mid-Term Outcomes
Source: J Cardiovasc Dev Dis. 2026 Feb 21;13(2):101. doi: 10.3390/jcdd13020101 (PMC12940880; doi:10.3390/jcdd13020101)

Figure S1. Distribution of standardized mean differences for baseline variables before and after propensity score matching.(A,B,C)

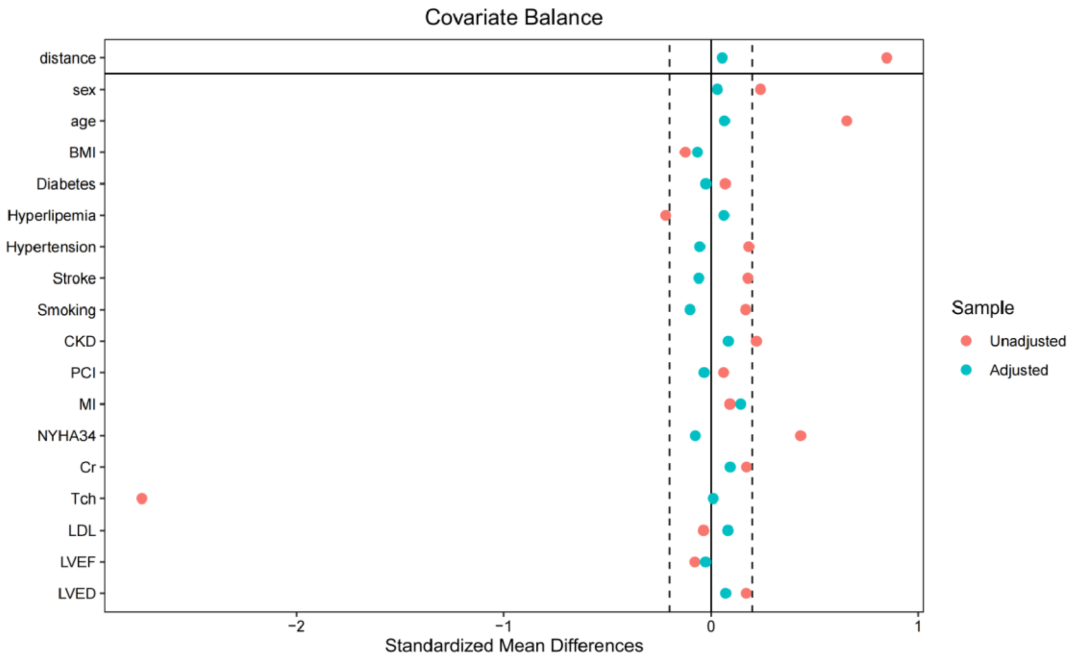

A: CKD, chronic kidney disease; PCI, percutaneous coronary intervention; MI, myocardial infarction; NYHA 34, New York Heart Association Functional Classification III-IV; Cr, serum creatinine; Tch, total cholesterol; LDL, low-density lipoprotein cholesterol; LVEF, left ventricular ejection fraction; LVEDD, left ventricular end-diastolic diameter.

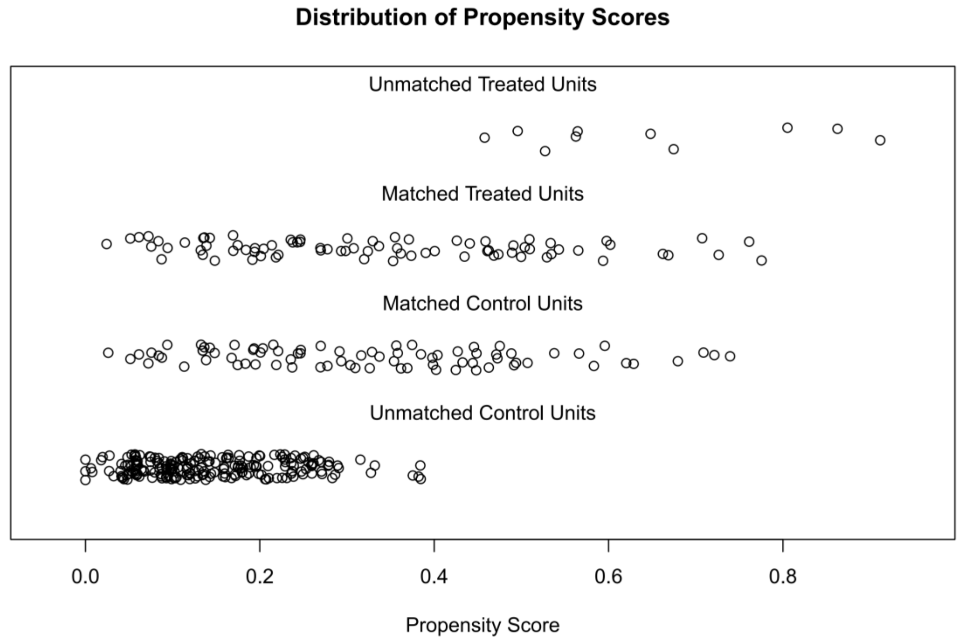

B: Treated units is AXA group；control units is AOR group.

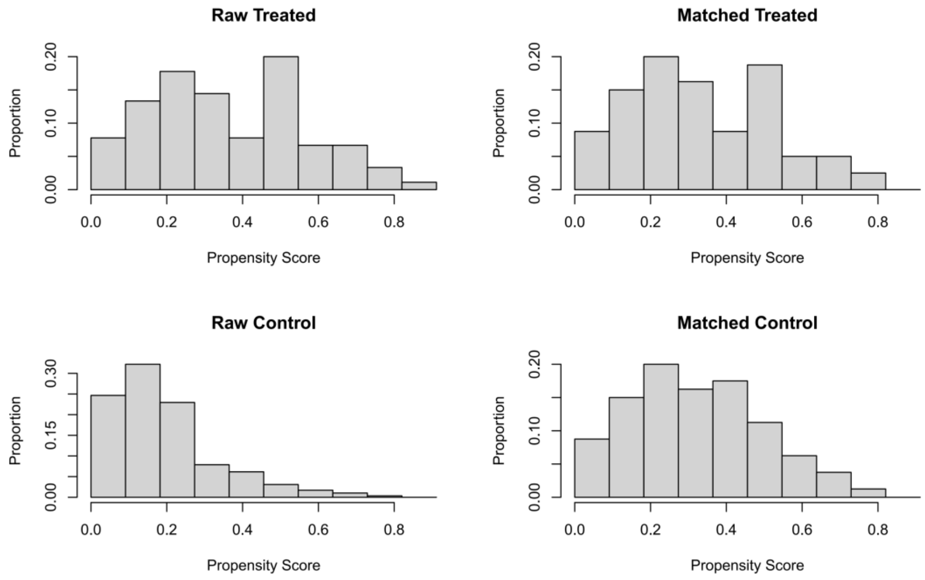

C: Treated units is AXA group； control units is AOR group.

Figure S2. Kaplan–Meier curves for mid-term follow-up outcomes before PSM. A, overall survival curve; B: freedom from MACCEs. The shaded areas represent 95% confidence intervals. P values were derived from Cox proportional hazards models adjusted for age and cardiac function.

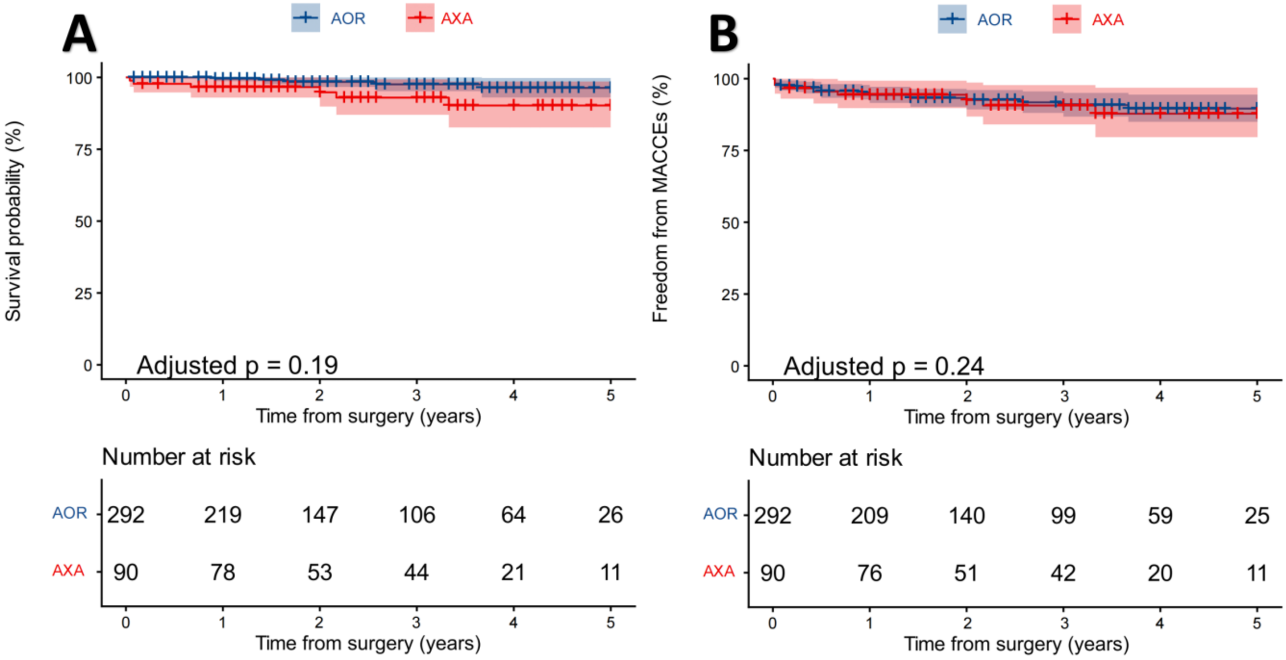

Supplement: Supplementary file 1 [file jcdd-13-00101-s001.zip › jcdd-4150166-supplementary.pdf]
